# Supplementary material for: Embryonic exposure to fentanyl induces behavioral changes and neurotoxicity in zebrafish larvae
Source: PeerJ. 2022 Dec 15;10:e14524. doi: 10.7717/peerj.14524 (PMC9760023; doi:10.7717/peerj.14524)
Supplement: Supplemental Information 3 [file peerj-10-14524-s003.docx]

**Table S1. Primes used.**

| Genes | Sequences | Specific temperature | Accession  number |
| --- | --- | --- | --- |
| β-actin-F | AGGTCATCACCATCGGCAAT | 59.45 | NM_131031.2 |
| β-actin-R | GATGTCCACGTCGCACTTCA | 60.67 |  |
| bdnf-F | TCGAAGGACGTTGACCTGTATG | 60.09 | [NM_001308649.1](https://www.ncbi.nlm.nih.gov/entrez/viewer.fcgi?db=nucleotide&id=821325130) |
| bdnf-R | TGGCGGCATCCAGGTAGT | 60.69 |  |
| c-fos-F | TGAAACTGACCAGCTTGAGGAT | 59.63 | [NM_205569.1](https://www.ncbi.nlm.nih.gov/entrez/viewer.fcgi?db=nucleotide&id=45387566) |
| c-fos-R | GTGTGCGGCGAGGATGAA | 60.43 |  |
| npas4a-F | ATGGGTCT GGTTTACATGG | 54.39 | [XM_688922.9](https://www.ncbi.nlm.nih.gov/entrez/viewer.fcgi?db=nucleotide&id=1207120776) |
| npas4a -R | CTTGTCTGGGTTGAGAGGAAC | 58.23 |  |
| egr1-F | CTAAGATCCACATGCGGCAGAAGG | 63.58 | [NM_131248.1](https://www.ncbi.nlm.nih.gov/entrez/viewer.fcgi?db=nucleotide&id=18858600) |
| egr1-R | AGTAGCAGGAGTTGACTGGAGACG | 63.67 |  |
| btg2-F | GGCGCTCTCAGAACACTACC | 60.46 | [NM_130922.2](https://www.ncbi.nlm.nih.gov/entrez/viewer.fcgi?db=nucleotide&id=425854824) |
| btg2-R | CTCACCGATGCGGTAGGATA | 58.76 |  |
| ier2a-F | GGAACAATGTGACAGCCGGT | 60.89 | [NM_001142583.3](https://www.ncbi.nlm.nih.gov/entrez/viewer.fcgi?db=nucleotide&id=1226253746) |
| ier2a-R | ACTTCTCGTCGAAGTACCGC | 59.83 |  |
| vgf-F | CAGGATTCCCGGTTCACACA | 59.96 | [XM_003198950.5](https://www.ncbi.nlm.nih.gov/entrez/viewer.fcgi?db=nucleotide&id=1207181248) |
| vgf-R | TGTTTCTGTCTGTTGTCACTGGA | 60.06 |  |
